# Association of circulating BMP9 with coronary heart disease and hypertension in Chinese populations

**DOI:** 10.1186/s12872-019-1095-2

**Published:** 2019-05-30

**Authors:** Rui Liu, Wenjing Hu, Xiaoqiang Li, Danlan Pu, Gangyi Yang, Hua Liu, Minghong Tan, Danping Zhu

**Affiliations:** 1 Department of Endocrinology, Chongqing Traditional Chinese Medicine Hospital, Chongqing, 400011 China; 2 Department of Endocrinology, the Second Affiliated Hospital Chongqing Medical University, Chongqing, China; 3 Chongqing Prevention and Treatment Hospital for Occupational Diseases, Chongqing, China; 4 Department of Clinical Laboratory, Children’s Hospital of Chongqing Medical University, Chongqing, China; 5 Department of Endocrinology, Pepople’s Hospital of Chongqing Banan District, Chongqing, China; 6 Department of Pediatrics, University of Mississippi Medical Center, 2500 North State Street, Jackson, MS 39216-4505 USA

**Keywords:** BMP9, Cytokine, Cardiovascular disease, Hypertension

## Abstract

**Background:**

Bone morphogenetic protein9 (BMP9) has been reported to have a role in vascular development. However, there is still a lack of information regarding the association between circulating BMP9 levels and cardiovascular disease in humans. The goal of this study is to measure circulating BMP9 concentrations in patients with essential hypertension (HTN), coronary heart disease (CHD) and HTN + CHD, and evaluates the relationship between circulating BMP9 and these cardiovascular diseases.

**Methods:**

A total of 417 individuals were recruited for this cross-sectional study from June 2015 to December 2017. These subjects were screened for HTN and CHD. Circulating BMP9 concentrations were measured by ELISA.

**Results:**

Circulating BMP9 concentrations were significantly low in HTN, CHD and HTN + CHD individuals relative to those of the healthy individuals. Circulating BMP9 correlated negatively with SBP, FIns and HOMA-_IR_ in HTN patients and correlated negatively with FBG and 2 h-BG in CHD patients. In both HTN and CHD patients, circulating BMP9 correlated negatively with BMI, WHR, FAT%, BP and TG. Multivariate logistic regression analysis showed that circulating BMP9 levels were associated with HTN, HTN + CHD and CHD. Individuals with low quartile of circulating BMP9 had a significantly high risk of HTN or/and CHD as compared with those in high quartile.

**Conclusions:**

BMP9 is likely to be a biomarker for cardiovascular disease in humans, and it may play a role in the progression of cardiovascular disease.

**Trial registration:**

ChiCTR-OPC-14005324.

**Electronic supplementary material:**

The online version of this article (10.1186/s12872-019-1095-2) contains supplementary material, which is available to authorized users.

## Background

Essential hypertension (HTN) and coronary heart disease (CHD) are two common diseases in cardiovascular clinics and their major risk factors include genetic, dietary and mental factors [1–4]. Cardiovascular diseases (CVD) cause nearly one third of all deaths worldwide. Coronary heart disease (CHD) accounts for the largest proportion of CVD [5]. Hypertension, dyslipidaemia, obesity and insulin resistance (IR) lead to an increased risk of leaving individuals prone to develop CVD [6]. In those affected by CVD and hypertension are a major contributor to the disease burden [7]. Among Chinese adults aged 35–75 years, nearly half have hypertension, fewer than a third are being treated, and fewer than one in twelve are in control of their blood pressure [8], which lead to increasing risk for CVD [9].

HTN and atherosclerosis are tightly linked metabolic disorders. HTN and CHD are growing global health problems that impact healthcare cost, quality of life and lifespan. Recent years, it has been found that cytokines secreted by hepatocytes, adipocytes and myocytes are signaling protein or extracellular polypeptide. They play an important role in the regulation of inflammation and insulin resistance (IR). Recently, a number of cytokines, such as members of bone morphogenetic proteins (BMPs) superfamily, has been reported to be involved in atherosclerotic lesions and implicated in the pathogenesis of CHD and HTN [10–13].

BMPs are a sub-classification of the transforming growth factor-β (TGF-β) superfamily. BMPs are secreted proteins and are characterized by their ability to induce ectopic bone formation [14, 15]. Recently, BMPs have been shown to be multifunctional cytokines and to have important roles in adipocyte differentiation, energy balance [16, 17] inducing the browning of adipocytes and promoting thermogenesis [18], preventing the formation of lymphatic vessel and human vascular disease [19].

BMP9 and BMP10 have been reported to play an important role in vascular development [20]. In addition, these two members of the BMP family can bind with high affinity to the endothelial-specific receptor activating receptor-like kinase 1 (ALK1) that is involved in vascular diseases [21]. As a secretory protein, BMP9 is also expressed in hepatocytes and secreted into the blood. Circulating BMP9 has also been shown as an important factor to maintain specific endothelial function [22]. In addition, BMP9 and its responsive genes have been reported to regulate vascular endothelial differentiation, promote angiogenesis, inhibit arteriosclerosis, and prevent vascular endothelial cell death [23]. Taken together, all these studies suggest that BMP9 may play an important role in the occurrence and development of vascular diseases, such as CHD and HTN. However, there is still a lack of report regarding the association between serum BMP9 and cardiovascular disease in human.

This study was designed as a cross-sectional cohort study to investigate the changes of circulating BMP9 levels in patients with cardiovascular diseases and its clinical significance. Therefore, we hypothesized that in CHD and HTN patients, circulating BMP9 levels were significantly altered and associated with metabolic disorders and vascular lesions.

## Methods

### Study design

This is a cross sectional study involving 417 individuals including 78 CHD patients (CHD group), 131 with HTN patients, 87 patients with hypertension complicated with coronary heart disease (CHD) and 121 healthy individuals were recruited in this study from June 2015 to December 2017. Circulating levels of BMP9 were measured in serum samples. The project mainly aimed to measure circulating BMP9 concentrations in patients with essential hypertension (HTN), coronary heart disease (CHD) and HTN + CHD. Further to evaluate the relationship between circulating BMP9 and these cardiovascular diseases. CHD patients were hospitalized with chest discomfort or pain and thus requires investigation with coronary CT angiography CHD was diagnosed by positive coronary angiography or angioplasty (an angiographic evidence of at least one 50% diameter stenosis in one or more coronary arteries) [24–26]. The degree of coronary atherosclerosis depends on the number of vessels in the lesion as a graded variable with significant stenosis. The Gensini score was determined according to the number of segments of stenotic coronary artery [27]. The diagnosis of HTN was based on WHO criteria [systolic blood pressure (SBP) ≥ 140 mmHg and/or diastolic blood pressure (DBP) ≥ 90 mmHg], and was confirmed after three visits. Secondary hypertension is determined by clinical, biochemical, hormonal measurements or/and imaging examination. Inclusion criteria for study population included following: 1) age 35–75 years; 2) body mass index (BMI) 17–35 kg/m^2^. Exclusion criteria included following: 1) acute myocardial infarction; 2) secondary hypertension; 3) type 2 diabetes mellitus (T2DM); 4) lung, liver and kidney disease; 5) cancer; 6) other known major diseases. Individuals with complication were eliminated, such as renal insufficiency, stroke, myocardial infarction and heart failure, etc. 121 age-matched healthy individuals, who had no clinical evidence of any diseases, no taking any medications, and had no family history of T2DM, HTN and CHD, were recruited and were used as the controls. These healthy individuals were recruited from routine medical check-up including coronary CT angiography. CHD in these subjects were excluded by CT coronary angiography (coronary CTA). The study was conducted in accordance with the Declaration of Helsinki, and was approved by the Human Research Ethics Committee of Chongqing Medical University (CHICTR- OCC- 13003185).

### Data collection

Anthropometric data were collected by a trained dietician in all participants. Body mass index (BMI) was calculated as weight in kilograms divided by squared height in meters (kg/m^2^). Waist circumference (WC) was measured midway between the lowest rib and the superior border of iliac crest on midaxillary line. The waist-to-hip ratio (WHR) was calculated by the same researcher. The percentage of body fat (FAT%) was measured by bioelectrical impedance (BIA-101; RJL Systems). Blood pressure (BP) was measured in all participants at least for the rest of 15 min. Blood samples were collected after an 8-10 h fasting and stored at − 80 °C for further measurements. Blood glucose and HbA1c were immediately measured by the glucose oxidase method and anion-exchange HPLC respectively. The homeostasis model assessment of insulin resistance (HOMA-_IR_) was calculated by the following equations: HOMA-_IR_ = fasting insulin (FIns, mU/mL) × fasting blood glucose (FBG, mmol/L) / 22.5 [28]. Insulin was measured by RIA using an ELISA kit. Free fatty acids (FFAs), total cholesterol (TC), triglyceride (TG), low-density lipoprotein cholesterol (LDL-C) and high-density lipoprotein cholesterol (HDL-C) were measured with a commercial kit as previous reported [29].

Circulating levels of BMP9 were measured in serum samples in duplicate with an ELISA kit for human BMP9 according to the manufacturer’s protocol (R&D Systems, Catalogue number #DY3209). The detection line was < 15.60 pg/mL. The intra- and inter-assay coefficients of variation were *<* 5 and *<* 10% respectively. The linear range was 15.6–1000 pg/ml. The assay has high sensitivity and excellent specificity for detection of human BMP9 with no cross-reactivity or interference between human BMP9 and other BMPs in circulation.

### Statistical analysis

All statistical analyses of this study were performed by A SPSS version 22.0 (SPSS Inc., Chicago, IL). A Kolmogorox-Smirnov test was performed for examining the distribution of data. ANOVA, paired- or unpaired t-test were used for comparison between groups. We used partial correlation coefficients and multivariate regression analyses to examine the association between circulating BMP9 and other variables, respectively. The multivariate logistic regression was used for analyzing the association of BMP9 with HTN or CHD. The Cochran-Armitage trend and row mean scores test were performed to assess the tendency of BMP9 concentration associated with HTN and CHD. The cut-off point of BMP9 level for predicting CHD were given by Receiver operating characteristics (ROC) curves. Sample size was calculated using the following equations: N = [Zα/2 σ/εμ]2 (σ, standard; μ, mean; Zα/2 = 1.96, α = 0.05, ε = 4%). All data were shown as mean ± SD or median (interquartile range). *P* < 0.05 were considered significant.

## Results

### Characteristics in study populations

The clinical characteristics of study population were shown in Table 1. The individuals with HTN had higher WHR, BMI, FAT (%), BP, TG, FIns and HOMA-_IR_ than control individuals (*P* < 0.05 or *P* < 0.01). In CHD patients, WHR, TG, 2-h blood glucose after glucose overload (2 h-BG), FIns, HbA1c and HOMA-_IR_ were significantly higher, while FFA was lower compared with healthy controls (*P* < 0.05 or *P* < 0.01). In addition, in patients with HTN + CHD, WHR, BMI, FAT (%), BP, TG, FIns, 2 h-BG, HbA1c and HOMA-_IR_ were significantly higher, whereas FFA was markedly lower relative to those of controls (*P* < 0.05 or *P* < 0.01; Table 1).

**Table 1:** Main clinical features and circulating BMP9 levels in the study population

Variable	Controls (*N* = 121)	HTN (*N* = 131)	HTN + CHD (*N* = 87)	CHD (*N* = 78)
Age (yr)	50.5 ± 11.7	51.5 ± 10.8	51.9 ± 10.5	52.4 ± 10.8
WHR	0.88 ± 0.08	0.90 ± 0.05*	0.92 ± 0.06*	0.93 ± 0.05**
BMI (kg/m^2^)	23.8 ± 3.4	25.1 ± 3.2*	24.8 ± 3.3*	23.9 ± 3.1
FAT (%)	28.7 ± 7.3	31.7 ± 7.0**	31.4 ± 6.9**	27.1 ± 5.9
SBP (mmHg)	117.7 ± 13.0	147.5 ± 23.1**	137.8 ± 17.9**	120.7 ± 10.5
DBP (mmHg)	74.4 ± 8.9	84.4 ± 13.6**	80.0 ± 10.9**	73.5 ± 7.3
TC (mmol/L)	4.52 ± 1.16	4.68 ± 0.90	4.47 ± 1.21	4.43 ± 1.41
TG (mmol/L)	1.22 (0.83–1.61)	1.51 (1.07–1.97)**	1.51 (1.07–2.54)**	1.28 (0.92–2.19)**
LDL-C (mmol/L)	2.74 ± 0.86	2.69 ± 0.76	2.96 ± 4.11	2.57 ± 1.02
HDL-C (mmol/L)	1.29 (1.04–1.54)	1.15 (1.00–1.31)	1.19 (0.95–1.23)	1.21 (0.88–1.28)
FFA (μmol/L)	0.53 (0.37–0.64)	0.56 (0.40–0.76)	0.41 (0.29–0.58)**	0.45 (0.33–0.63)**
FBG (mmol/L)	5.28 ± 0.53	5.29 ± 0.41	5.18 ± 0.45	5.18 ± 0.39
FIns (mU/L)	9.89 (5.38–15.10)	21.9 (18.4–30.0) **	13.7 (7.4–21.5)**	12.4 (6.5–19.8)**
2 h-BG (mmol/L)	6.29 ± 1.07	6.55 ± 0.84	6.62 ± 0.66*	6.85 ± 1.03*
HbA1c (%)	5.65 ± 0.40	5.69 ± 0.39	5.73 ± 0.27*	5.71 ± 0.31*
HOMA-IR	2.45 (1.50–3.60)	5.04 (4.16–6.83)**	3.16 (1.61–4.88)**	2.71 (1.60–4.28)*
BMP9 (ng/L)	123.3 (45.5–178.6)	53.2 (31.8–62.8) **	52.9(27.2–74.7) **	55.0 (24.9–71.4)**

HTN, essential hypertension; CHD, coronary heart disease; WHR, waist hip ratio; BMI, body mass index; FAT%, the percentage of fat in vivo; SBP, systolic blood pressure; DBP, Diastolic blood pressure; TC, total cholesterol; TG, triglyeride; LDL-C, Low-density lipoprotein cholesterol; HDL-C, High-density lipoprotein cholesterol; FFA, free fatty acid; FBG, Fasting blood glucose; FIns, fasting insulin; 2 h-BG, 2-hourblood glucose after glucose overload; HOMA-IR, homeostasis model assessment of insulin resistance. Data are mean ± SD or median (interquartile range). *Data are mean ± SE, Adjustment for age, gender, BMI. *P < 0.05 or **P < 0.01 compared with controls

### Circulating BMP9 levels in study population and its correlation with clinical and biochemical parameters

**Fig. 1:**
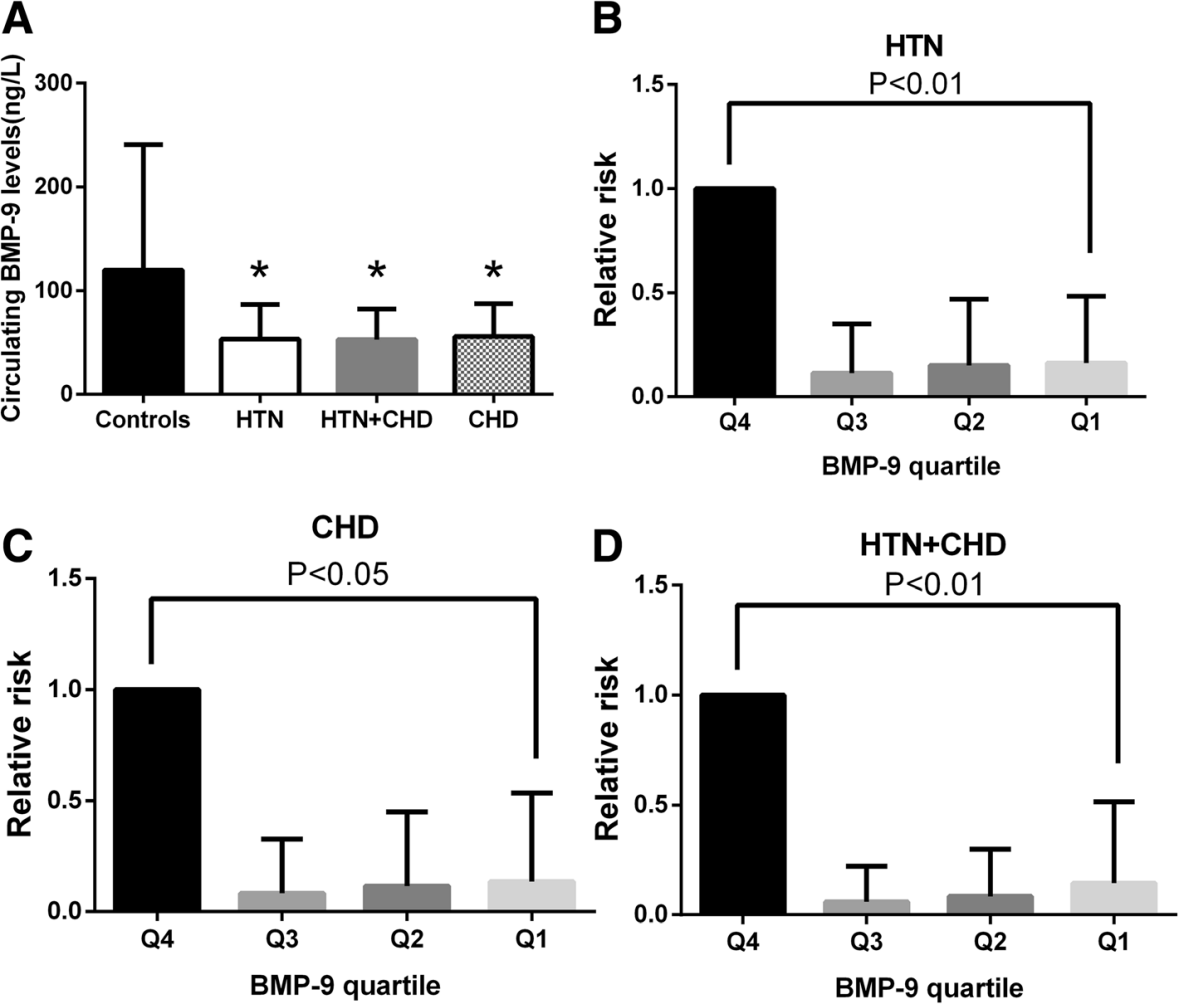
Analysis of serum BMP9 by different statistical approaches. **a**, Circulating BMP9 levels in HTN, CHD and HTN + CHD patients; **b**, The odds ratio of having HTN in different quartile of BMP9; **c**, The odds ratio of having CHD in different quartile of BMP9; **d**, The odds ratio of having HTN + CHD in different quartile of BMP9. *P < 0.05, **P < 0.01 vs. controls or quartile 4

In the current study, we found that circulating BMP9 levels were significantly reduced in HTN, CHD and patients with both HTN and CHD, when compare with healthy controls (all *P* < 0.01, Fig. 1a and Table 1). After adjusting gender and age, these differences are still significant. Among the three groups of patients, the BMP9 levels in HTN + CHD group were the lowest, but there was no significant difference between the three groups. In partial correlation analysis, we found that the circulating BMP9 correlated negatively with SBP, FIns and HOMA-_IR_ in HTN patients. In both HTN and CHD patients, circulating BMP9 correlated negatively with BMI, WHR, FAT%, BP and TG (*P* < 0.05 or *P* < 0.01; Table 2). Finally, in CHD patients, circulating BMP9 correlated negatively with FBG and 2 h-BG (*P* < 0.05 or *P* < 0.01; Table 2). These associations remained statistically significant after adjustment for age and sex. In multiple regression analysis of variables, circulating BMP9 levels were independently related to SBP and FFA in HTN patients (both *P* < 0.01), BMI and FFA in HTN + CHD patients (both *P* < 0.01) as well as 2 h-BG in CHD patients (*P* < 0.05, Table 2).

**Table 2:** Linear regression analysis of variables associated with circulating BMP9 levels in the study population

	HTN			HTN + CHD				CHD				
	Simple		Multiple		Simple		Multiple		Simple		Multiple	
Variable	R	*P*	β	*P*	R	*P*	β	*P*	R	*P*	β	*P*
Age (yr)	−0.086	0.201			0.289	<0.01			−0.021	0.856		
BMI (kg/m^2^)	0.089	0.190			−0.100	<0.001	−9.033	<0.001	−0.005	0.966		
WHR	−0.049	0.461			−0.333	<0.01			−0.022	0.847		
FAT (%)	−0.085	0.205			−0.515	<0.001			0.158	0.166		
SBP (mmHg)	−0.219	0.006	−0.004	<0.001	− 0.345	<0.001			−0.165	0.149		
DBP (mmHg)	−0.085	0.205			−0.779	<0.001			0.078	0.497		
TG (mmol/L) ^**a**^	0.081	0.231			−0.303	<0.01			−0.142	0.214		
TC (mmol/L)	0.066	0.330			0.054	0.621			0.026	0.820		
HDL-C (mmol/L)^**a**^	−0.003	0.965			0.193	0.076			0.085	0.459		
LDL-C (mmol/L)	0.140	0.341			0.092	0.397			0.156	0.173		
FFA (mmol/L) ^**a**^	0.136	0.012	0.203	<0.001	0.634	<0.001	0.114	<0.001	0.158	0.166		
FBG (mmol/L)	0.118	0.076			−0.096	0.379			−0.232	< 0.05		
2 h-BG (mmol/L)	−0.134	0.051			0.009	0.938			−0.10	< 0.01	−7.394	<0.05
FIns (mU/L) ^**a**^	−0.148	0.015			0.211	0.052			0.120	0.297		
HbA1c(%)	−0.567	0.234			0.017	0.873			−0.030	0.792		
HOMA-IR^**a**^	−0.135	0.030			0.195	0.073			0.138	0.228		

In multiple linear regression analysis, values included for analysis were age, sex, BMI, WHR, FAT, TG, HDL,TC,HOMA-IR,2 h-BG, FFA. ^a^Log transformed before analysis

### Association of circulating BMP9 with HTN and CHD

To investigate the relationship between circulating BMP9 and HTN and CHD, we performed multivariate logistic regression analysis. The results showed that circulating BMP9 levels were associated with HTN, HTN + CHD and CHD, even after controlling for anthropometric variables, age, gender, FAT%, blood pressure and lipid profile (Table 3). In addition, to investigate the relationship between BMP9 titer stratification and HTN and CHD, we also performed Row mean scores differ and Cochran-Armitage trend test in all study population. The results revealed that the decreasing levels of BMP9 showed a significant linear trend and were independently associated with HTN and CHD, when the concentration was analyzed (Table 4).

**Table 3:** Association of circulating BMP9 with HTN and CHD in fully adjusted models

	HTN			HTN + CHD			CHD		
	OR	95%CI	*P*-value	OR	95%CI	*P*-value	OR	95%CI	*P*-value
Model 1	0.983	0.978–0.989	<0.001	0.984	0.976–0.992	<0.001	0.988	0.981–0.996	<0.01
Model 2	0.983	0.978–0.989	<0.001	0.987	0.979–0.995	<0.001	0.988	0.980–0.996	<0.01
Model 3	0.983	0.978–0.989	<0.001	0.990	0.982–0.998	<0.05	0.988	0.980–0.996	<0.01
Model 4	0.985	0.978–0.991	<0.001	0.984	0.974–0.994	<0.001	0.987	0.979–0.995	<0.01

Model1, adjusted age, gender; Model2, adjusted age, gender, WHR; Model3, adjusted age, gender, WHR BMI, FAT, FBG; Model 4, adjusted age, gender, WHR BMI, FAT, FBG, lipid profile. Results of multivariate logistic regression analysis are presented as the odds ratio (OR) of being in HTN, HTN + CHD and CHD status increase in circulating BMP9

**Table 4:** Row mean scores differ and Cochran-Armitage trend analysis of the impact of circulating BMP9 levels on HTN and CHD

	HTN		HTN + CHD		CHD	
Model adjusted	*X* ^2^	*P*-value	*X* ^2^	*P*-value	*X* ^2^	*P*-value
Row Mean Scores Test	55.260	<0.001	7.451	<0.01	5.275	<0.05
Cochran-Armitage Trend Test	3.6421	<0.001	2.736	<0.01	2.303	<0.05

Values shown are cut-offs of circulating BMP9 levels of all subjects. Adjusted for age, sex, BMI, WHR, blood pressure, TG, TC, LDL-C and HDL-C

### The relative risk of prevalent of cardiovascular diseases for circulating BMP9 levels

According to the concentration of BMP9 in HTN, CHD and HTN + CHD patients, it is divided into quartile (quartile 1, < 31.80 ng/L, quartile 2, 31.81–46.44 ng/L, quartile 3, 46.45–62.80 ng/L, quartile 4, > 62.80 ng/L for HTN; quartile 1, < 27.24 ng/L, quartile 2, 27.25–42.25 ng/L, quartile 3, 42.26–74.71 ng/L, quartile 4, > 74.71 ng/L for HTN + CHD and quartile 1, < 24.98 ng/L, quartile 2, 24.99–54.95 ng/L, quartile 3, 54.96–70.86 ng/L, quartile 4, > 70.86 ng/L, respectively). As shown in Fig. 1b-d, individuals in low quartile of circulating BMP9 had a significantly high risk of HTN or CHD or both HTN and CHD compared with those of high quartile. These changes still exist, even after the adjustment of age, sex, BMI and WC.

To evaluate the relationship between BMP9 and CHD or HNT, respectively, circulating BMP9 concentrations were further stratified. We found that each stratified concentration was correlated with the risk of CHD or HNT, respectively. With per standard deviation equivalent (1-SD) decrease of BMP9 concentrations, the risk of CHD or HTN increased significantly (Additional file 1: Table S1).

**Fig. 2:**
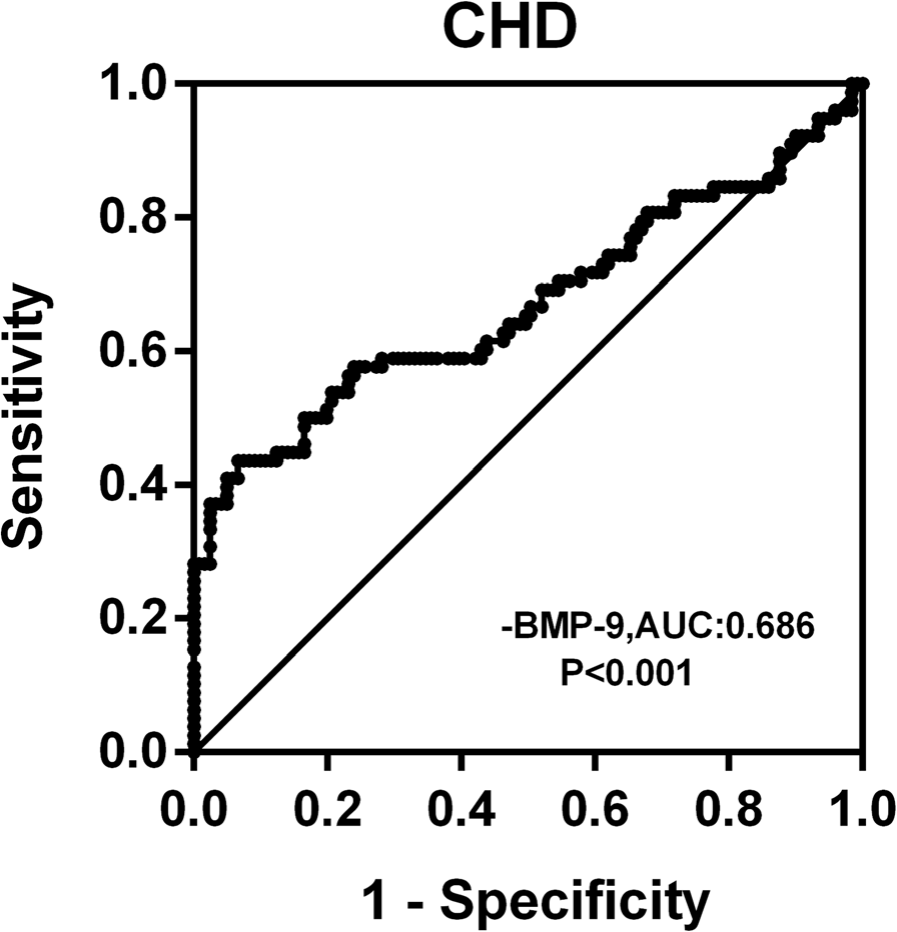
ROC curve analysis was performed for the prediction of CHD

To explore the predictive value of circulating BMP9 for CHD, we performed the ROC curves analysis. This analysis showed that the area under the ROC curves (AUC) was 0.686 (*P* < 0.001) with a sensitivity of 62.8% and specificity of 44.9% for CHD (Fig. 2), and the best cutoff values for circulating BMP9 to predict CHD was 57.3 ng/L.

## Discussion

HTN is a common cardiovascular disease, and the causal factors include genetic, dietary and mental factors [30–32]. The main risk of HTN is a continuous increase in blood pressure, thereby increasing the burden of the left ventricle and eventually leading to CHD [33]. Therefore, it is important to explore the common biomarkers between HTN and CHD.

This cross-sectional study was performed in a community-based middle to older aged Chinese population. Up to now, few studies have reported circulating BMP9 levels in both HTN and CHD patients and the association of circulating BMP9 with HTN and CHD risk in humans. In the current study, we found that fasting BMP9 levels were lower in HTN, CHD or HTN + CHD patients than those of healthy controls. These results are similar to our previous report in T2DM patients [34]. Therefore, current and previous results indicated that BMP9 could have a role in linking metabolic disorder and arterial stiffness, and have an impact on the pathophysiology of IR and arteriosclerosis-related diseases. Although the nature of the current study does not permit us to determine the cause of decreased circulating BMP9 in CHD and HTN patients, we speculate that the decrease of BMP9 in patients with cardiovascular disease might be a defensive response to metabolic stress and arteriosclerosis. Therefore, whether increasing circulating BMP9 level can improve lipid metabolism, stabilize arterial plaque and improve vascular endothelial function should be further studied.

In the current study, circulating BMP9 correlated negatively with FBG and 2 h-BG in CHD patients, while 2 h-BG was an independently related factor influencing circulating BMP9 levels. Therefore, in CHD patients, circulating BMP9 levels were mainly affected by blood glucose levels, suggesting an association between BMP9 and glucose metabolism. In patients with both HTN and CHD, circulating BMP9 correlated negatively with BP, parameters of adiposity (BMI, WHR and FAT %) and parameters of fat metabolism (TG and FFA). BMI and FFA were independently related factors for circulating BMP9 levels. Therefore, circulating BMP9 was mainly impacted by obesity and the disorder of lipid metabolism in these patients.

The analyses employing 3 quartiles of circulating BMP9 demonstrated that patients with lower quartiles of BMP9 were more likely to develop HTN, CHD and HTN + CHD when compared to those in the highest quartile. To assess the diagnostic capacity of circulating BMP9 in CHD and the coexistence of CHD, ROC curve analysis was performed. The ROC analysis further showed that BMP9 was associated with CHD. BMP9 could be a discriminative performance measure as an indicator of cardiovascular disease. The cut-off point of ROC curve indicated sensitivity and specificity values between 40 and 60%, which minimizes false-positive and false-negative cases.

Therefore, circulating BMP9 has certain clinical value in the diagnosis of cardiovascular diseases. Based on these results, we postulate that BMP9 may serve as a biomarker for the progress of cardiovascular diseases.

### Limitations

1). This was a cross-sectional study with a single center on Chinese patients, it can’t determine a cause-effect relationship between circulating BMP9 and HTN as well as CHD; 2) Our study did not examine the sources of BMP9; 3) The size of the sample is relatively small considering that it is divided into four study groups, one independent from the other, and each with a different number of patients. Therefore, clinical application may be limited; 4) Our study was based on single measurements of serum BMP9, which may not reflect the changes of circulating BMP9 over time. Therefore, it would be interesting to take a new measurement of BMP9 after a few months; 5) As the population studied is exclusively Chinese, it cannot be applied to the general population. Therefore, serial alternation of serum BMP9 should be measured at different stages of these patients to clarity the role of BMP9 at the onset of HTN and CHD. Finally, the study individuals were from Chinese populations which have similar lifestyles. Thus, these data may not be directly applicable to other races.

## Conclusions

Our study demonstrated that circulating BMP9 levels are decreased in HTN, CHD and HTN + CHD individuals, and circulating BMP9 correlated with the prevalence rate of cardiovascular diseases. We postulate that the risks of HTN, CHD and HTN + CHD increase as circulating BMP9 concentration increase. Therefore, our data suggest that BMP9 plays a role in the pathophysiology of HTN and CHD, and circulating BMP9 is likely to be a biomarker for the progress of HTN, CHD and HTN + CHD.

## Additional file

Additional file 1: **Table S1**. The risk of prevalent of CHD or HNT according to quartiles for serum BMP9 concentrations. (DOCX 16 kb)

## Abbreviations

2 h-BG: 2-h blood glucose after glucose overload
BMI: Body mass index
BP: Blood pressure
FAT %: The percentage of body fat
FBG: Fasting blood glucose
FFA: Free fatty acid
Fins: Fasting insulin
HOMA-_IR_: Homeostasis model assessment of insulin resistance
SBP: Systolic blood pressure
TG: Triglyceride
WHR: Waist hip ratio
