# Supplementary material for: Association of circulating BMP9 with coronary heart disease and hypertension in Chinese populations
Source: BMC Cardiovasc Disord. 2019 May 30;19:131. doi: 10.1186/s12872-019-1095-2 (PMC6543594; doi:10.1186/s12872-019-1095-2)
Supplement: Supplementary file 1 — Table S1. The risk of prevalent of CHD or HNT according to quartiles for serum BMP9 concentrations. (DOCX 16 kb) [file 12872_2019_1095_MOESM1_ESM.docx]

**Table S1 The risk of prevalent of CHD or HNT according to quartiles for serum BMP9 concentrations.**

|  |  | **Q4** | | **Q3** | ***p*-value** | **Q2** | ***p*-value** | **Q1** | ***p*-value** | | **1 SD of BMP9** |
| --- | --- | --- | --- | --- | --- | --- | --- | --- | --- | --- | --- |
| **HNT** | **Model1** | 1 | 0.145(0.064-0.325) | | <0.001 | 0.104(0.045-0.238) | <0.001 | 0.172(0.077-0.382) | | <0.001 | 0.478(0.347-0.658) |
|  | **Model2** | 1 | 0.141(0.062-0.319) | | <0.001 | 0.104(0.045-0.239) | <0.001 | 0.174(0.078-0.388) | | <0.001 | 0.479(0.349-0.658) |
|  | **Model3** | 1 | 0.110(0.046-0.264) | | <0.001 | 0.095(0.040-0.227) | <0.001 | 0.164(0.071-0.380) | | <0.001 | 0.453(0.323-0.636) |
|  | **Model4** | 1 | 0.109(0.045-0.261) | | <0.001 | 0.095(0.040-0.227) | <0.001 | 0.164(0.071-0.379) | | <0.001 | 0.456(0.325-0.637) |
|  | **Model5** | 1 | 0.104(0.041-0.269) | | <0.001 | 0.121(0.047-0.310) | <0.001 | 0.191(0.077-0.474) | | <0.001 | 0.458(0.318-0.656) |
|  | **Model6** | 1 | 0.103(0.033-0.317) | | <0.001 | 0.146(0.048-0.447) | 0.001 | 0.141(0.048-0.415) | | <0.001 | 0.476(0.320-0.710) |
|  | **Model7** | 1 | 0.113(0.036-0.351) | | <0.001 | 0.150(0.048-0.469) | 0.001 | 0.162(0.055-0.483) | | <0.001 | 0.474(0.332-0.675) |
| **CHD** | **Model1** | 1 | 0.080(0.027-0.237) | | <0.001 | 0.161(0.054-0.477) | <0.001 | 0.120(0.041-0.354) | | <0.001 | 0.029(0.093-0.470) |
|  | **Model2** | 1 | 0.076(0.021-0.275) | | <0.001 | 0.148(0.041-0.531) | 0.003 | 0.120(0.034-0.424) | | <0.001 | 0.236(0.095-0.591) |
|  | **Model3** | 1 | 0.074(0.020-0.269) | | <0.001 | 0.143(0.040-0.513) | 0.003 | 0.115(0.032-0.408) | | <0.001 | 0.232(0.092-0.587) |
|  | **Model4** | 1 | 0.073(0.020-0.267) | | <0.001 | 0.144(0.040-0.517) | 0.002 | 0.117(0.033-0.414) | | <0.001 | 0.231(0.093-0.575) |
|  | **Model5** | 1 | 0.077(0.020-0.295) | | 0.002 | 0.125(0.033-0.473) | 0.002 | 0.124(0.033-0.463) | | <0.001 | 0.236(0.089-0.624) |
|  | **Model6** | 1 | 0.078(0.020-0.300) | | 0.002 | 0.128(0.034-0.485) | 0.002 | 0.123(0.032-0.468) | | <0.001 | 0.229(0.084-0.625) |
|  | **Model7** | 1 | 0.083(0.021-0.328) | | 0.036 | 0.116(0.030-0.451) | 0.002 | 0.136(0.035-0.535) | | <0.001 | 0.216(0.081-0.578) |

CHD: 1) Q1, ≤ 24.98 ng/L; 2) Q2, 24.99 - 54.95ng/L; 3) Q3, 54.96 - 70.86 ng/L; 4) Q4, > 70.86ng/L.

HNT: 1) Q1, ≤ 31.80ng/L; 2) Q2, 31.81 - 46.44ng /L; 3) Q3, 46.45 - 62.80ng/L; 4) Q4 > 62.80ng/L.

Model1, unadjusted; Model 2, Model 1+ Age, gender; Model 3, Model2+ BMI; Model4, Model3+ WHR; Model5, Model4+ WHR+ FAT%;

Model6, Model5+ SBP, DBP; Model7, Model6+ lipid profile; A two-tailed level of significance was established as *P* < 0.05.
